# Petunidin attenuates lipopolysaccharide-induced retinal microglia inflammatory response in diabetic retinopathy by targeting OGT/NF-κB/LCN2 axis

**DOI:** 10.1515/med-2025-1274

**Published:** 2025-09-06

**Authors:** Mengxi Yu

**Affiliations:** Department of Ophthalmology, Affiliated Wuxi Clinical College of Nantong University, Wuxi, 214000, Jiangsu, China; Department of Ophthalmology, Jiangnan University Medical Center (JUMC), Wuxi, 214000, Jiangsu, China; Department of Ophthalmology, Wuxi No.2 People’s Hospital, Wuxi, 214000, Jiangsu, China

**Keywords:** diabetic retinopathy, diabetes mellitus, lipopolysaccharide

## Abstract

**Background:**

Diabetic retinopathy (DR), as a frequent complication of diabetes mellitus, is a common cause of vision impairment and blindness. Microglial activation plays an important role in the pathological cascade of DR, and novel potential therapeutics is needed to interfere with this process. Petunidin (PET) is one of the most abundant natural anthocyanins with the biological activities of anti-inflammation, anti-cancer, and anti-microbial.

**Objective:**

The purpose of this study is to investigate the effect of PET against lipopolysaccharide (LPS)-induced retinal microglia inflammatory response as well as the underpinning mechanism.

**Methods:**

Cell viability was determined using MTT assay. Cytokines secretion was determined using ELISA assay. iTRAQ-based proteomic analysis was used for the identification of differentially expressed proteins. Western blot analysis, Co-IP and immunofluorescence analysis were applied in the study.

**Results:**

The results showed that PET pre-treatment significantly reduced LPS-induced cytokines secretion in BV2 cells and primary retinal microglia, as well as lipocalin 2 (LCN2) upregulation in BV2 cells by suppressing activation of *O*-GlcNAc modification and activation of NF‑κB. Further study revealed that PET inactivated NF‑κB by down-regulating OGT in BV2 cells, indicating that the protective effect of PET against LPS-induced retinal microglia inflammatory response was achieved by regulating OGT/NF-κB/LCN2 axis.

**Conclusion:**

Our findings may contribute to the potential clinical use of PET in treating DR and suggest OGT/NF-κB/LCN2 axis may be the potential therapeutic target of this disease.

## 1 Introduction

Diabetic retinopathy (DR), the major ocular complication of diabetes mellitus, becomes a significant global health issue [1]. Currently, anti-vascular endothelial growth factor therapy demonstrates remarkable clinical benefits in patients with later-stage DR; however, treating early-stage DR remains challenging [2,3]. Laboratory and clinical evidence indicate that before the onset of microvascular changes, inflammation, and retinal neurodegeneration may contribute to diabetic retinal damage in the early-stage of DR [4,5,6,7]. Further investigation of the underlying molecular mechanisms may provide targets for the development of new early interventions. Microglia, the defense and homeostatic cells of the immune system that originate from bone marrow progenitor cells, is involved in the pathological process of inflammation, oxidative stress, angiogenesis, and tissue healing processes in DR [8,9,10]. Therefore, to investigate the possible regulatory role of retinal microglial cells in early-stage DR may provide new targets and insights for early intervention of DR.

Increased evidence indicates that the natural compounds from fruits and vegetables, such as flavonoids, exert a diverse array of biological and pharmacological effects and are increasingly acknowledged as promising candidates for therapeutic applications [11,12]. Anthocyanins as a subclass of flavonoids demonstrate a spectrum of health-promoting benefits in human diseases, such as cancer, diabetes, obesity, and cardiovascular disease, due to their antioxidant properties [13,14,15]. Petunidin (PET) is a type of anthocyanin and exhibits anti-oxidative and anti-inflammatory properties and active sites through both *in silico* and in laboratory experiments [16,17]. Cai et al. reported that PET prevents cardiomyocytes apoptosis by inhibiting the production of ROS, reducing oxidative stress, and regulating the NOX4 signaling [18]. Liu et al. have reported that PET improves thyroid dysfunction by suppressing apoptosis, oxidative stress, and Th1/Th17 differentiation through regulation of the NOX4/PKM2 axis in HT mice [19]. However, the anti-inflammatory effect and molecular mechanism of PET’s pharmacological activity on DR has never been explored.

In the present study, using lipopolysaccharide (LPS)-stimulated BV2 microglia and primary retinal microglia (PRMC) as *in vitro* model due to the previous reports [20,21], we sought to examine whether PET has the protective effect against microglial dysfunction under diabetic stress and further investigate the underlying mechanism.

## 2 Materials and methods

### 2.1 Chemicals and reagents

PET chloride (C_16_H_13_O_7_Cl), SN50, and nuclear factor-κB (NF-κB) activator 1 were obtained from MCE (Shanghai, China). Reagents for cell culture were obtained from Life Technology (Waltham, MA, USA). The ELISA kits for TNF-α, IL-1β, and IL-18 were obtained from Solarbio Biotech (Beijing, China). Antibodies were obtained from Santa Cruz Biotechnology (Dallas, CA, USA) and Abcam (Cambridge, MA, USA) as follows: lipocalin 2 (LCN2) (cat. no. ab125075, Abcam, 1:1,000), p65 (cat. no. ab32536, Abcam, 1:1,000), *O*-GlcNAc (cat. no. ab308178, Abcam, 1:1,000), OGT (cat. no. ab177941, Abcam, 1:1,000), and GAPDH (cat. no. sc-365062, Santa Cruz, 1:500). Other chemicals and reagents were purchased from Beyotime Institute (Nantong, China) and Sangon Biotech (Shanghai, China).

### 2.2 Cell culture and treatment

The microglial cell line BV2 and HEK293T cells were obtained from the American Type Culture Collection (ATCC, Manassas, VA, USA). Cells were seeded at a density of 20,000 cells/cm² and cultured in DMEM medium supplemented with 10% fetal bovine serum (FBS), 100 U/mL penicillin, and 100 µg/mL streptomycin at 37°C with a humidified atmosphere of 95% air and 5% CO_2_.

PRMC was isolated from eyeball residues of an adult human donor in Wuxi No. 2 People’s Hospital, Jiangnan University Medical Center as previously reported [22,23]. The study was approved by the Ethics Committee (License NO. YL202121) and signed informed consent was obtained from the participant. Briefly, retinal tissues were dissected from eyeball remnants, minced with scissors, and thoroughly mixed with trypsin digestion solution. The tissue fragments were then filtered through a cell strainer and seeded into poly-lysine-coated flasks containing DMEM/F-12 medium supplemented with 15% FBS, 1% penicillin/streptomycin, and 25 ng/mL macrophage colony-stimulating factor (Beyotime, Nantong, China). After 1 week of culture, the cells were characterized through Iba-1 immunofluorescence staining before being used for subsequent experiments.

Following treatment with various concentrations of PET for 24 h, cells were primed with LPS at a concentration of 0.1 µg/mL for an additional 24 h. For cell transfection, pcDNA3.1-LCN2 or pcDNA3.1-OGT (Genescript, Nanjing, China) was introduced into the cells using Lipofectamine 2000 (Life Technologies) according to the manufacturer’s instructions.

### 2.3 Cell viability assay

MTT (3-(4, 5-dimethylthiazol-2-yl)-2, 5-diphenyltetrazolium bromide) assay was used to determine cell viability. After treatment, MTT solution (0.5 mg/mL, 100 μL) was added to the cell culture, which was further incubated for 3 h at 37°C. After removing the medium, DMSO (dimethyl sulfoxide, 150 μL) was added to the cell culture for 10 min with gentle shaking. The absorbance was detected with a microstrip reader (BioRad Laboratories, Hercules, CA, USA) at 490 nm wavelength.

### 2.4 ELISA analysis assay

The levels of IL-1β, TNF-α, and IL-18 in the medium of BV2 cells or PRMC with the indicated treatment were analyzed using ELISA kit according to the manufacturer’s instructions. The absorbance was measured at 450 nm using a microplate reader (BioRad Laboratories, Hercules, CA, USA).

### 2.5 Western blot analysis

Whole-cell extracts were prepared in lysis buffer as previously described [24]. The lysates were centrifuged, and the supernatants were collected. Protein concentrations were determined using the Bradford assay. Twenty micrograms of protein were separated by 10% SDS-PAGE and transferred to polyvinylidene fluoride membranes. The membrane was then blocked and incubated with the primary antibody overnight at 4°C. After being washed three times with TBST, the membrane was incubated with horseradish peroxidase-conjugated secondary antibody at 37°C for 2 h. The antibody binding was detected using an enhanced chemiluminescence detection kit.

### 2.6 iTRAQ-based proteomic analysis

Cells were harvested and lysed, and protein concentration was determined using the BCA assay. Subsequently, the iTRAQ-based proteomic analysis was divided into two phases. The preliminary experimental procedure included the following steps: protein extraction, protein quantification, proteolytic digestion, mass spectrometry, and database searching. The formal experiment was conducted based on the preliminary steps and comprised iTRAQ peptide labeling, fractionation, mass spectrometry analysis, and database comparison.

### 2.7 Dual-luciferase reporter assay

To evaluate the effect of p65 (NF-κB) on LCN2, HEK293T cells were plated in 6-well plates and co-transfected with either pcDNA3.1-vector or pcDNA3.1-p65, and either pGL3-LCN2 WT or pGL3-LCN2 MUT (Genepharma, Shanghai, China) using Lipofectamine 2000. Forty-eight hours after transfection, cells were collected and analyzed using the Dual-luciferase Reporter Assay System (Promega, Madison, WI, USA).

### 2.8 Co-immunoprecipitation (Co-IP) assay

Cell lysate was incubated with protein A/G agarose beads conjugated to antibody for 24 h at 4°C. The beads were subsequently washed three times with Tris-buffered saline (TBS), and the immunoprecipitated proteins were eluted using 2× SDS-PAGE sample buffer. The samples were then analyzed by western blot analysis.

### 2.9 Immunofluorescence analysis

Cells were fixed in 2% paraformaldehyde and permeabilized in 0.1% Triton X-100, followed by incubation with primary antibody and fluorescent-conjugated secondary antibody. Fluorescence was observed by Leica Microsystems fluorescence microscope (Wetzlar, Germany). DAPI was used to stain the nuclei.

### 2.10 Data analysis

Statistical analyses were performed with GraphPad Software (San Diego, CA, USA). All data were presented as the mean values ± SD for a minimum of three independent experiments in triplicate. All comparisons were made using one-way ANOVA followed by Tukey’s *post hoc* test, with significant differences determined as *p* < 0.05.

## 3 Results

### 3.1 PET attenuates LPS-induced inflammatory status in BV-2 cells and PRMC

**Figure 1:**
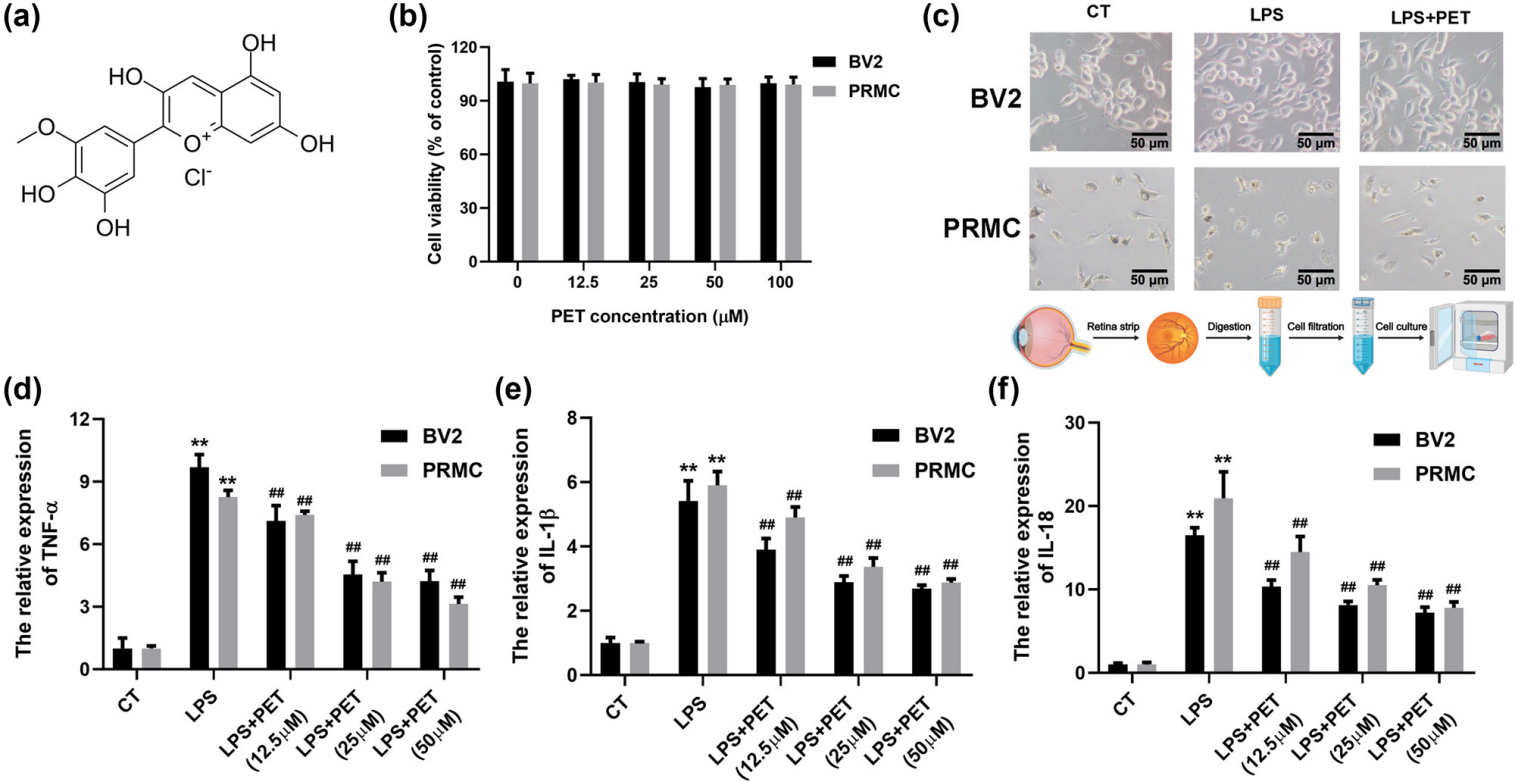
PET attenuated LPS-induced inflammatory status in BV-2 cells and PRMC. (a) The chemical structure of PET. (b) The effects of PET (0, 12.5, 25, 50, 100 µM) on cell viability of BV2 cells and PRMC were assessed by MTT assay. (c) The effects of PET (25 µM) on cell morphology of BV2 cells and PRMC upon LPS treatment (0.1 µg/mL) were assessed. (d) The effects of PET (0, 12.5, 25, 50 µM) on TNF-α of BV2 cells and PRMC upon LPS treatment (0.1 µg/mL) were assessed by ELISA assay. (e) The effects of PET (0, 12.5, 25, 50 µM) on IL-1β of BV2 cells and PRMC upon LPS treatment (0.1 µg/mL) were assessed by ELISA assay. (f) The effects of PET (0, 12.5, 25, 50 µM) on IL-18 of BV2 cells and PRMC upon LPS treatment (0.1 µg/mL) were assessed by ELISA assay. **p < 0.01 vs CT, ^##^ *p* < 0.01 vs LPS (0.1 µg/mL). CT: the control group.

To determine the effects of PET (Figure 1a) on inflammatory status induced by LPS stimulation, cell viability, cell morphology, and cytokines levels were assessed in BV2 cells and PRMC. As showed in Figure 1b and c, PET showed non-cytotoxic effects and attenuated LPS-induced cell morphology changes in BV-2 cells and PRMC. Then, the expression levels of TNF‑α, IL‑1β, and IL‑18 were strongly induced by LPS stimulation; however, PET pre-treatment significantly attenuated the pro-inflammatory effects of LPS (Figure 1d–f). The data indicated the robust anti-inflammatory effects of PET in LPS‑treated BV-2 cells and PRMC.

### 3.2 PET attenuates LPS-induced inflammation via downregulation of LCN2

**Figure 2:**
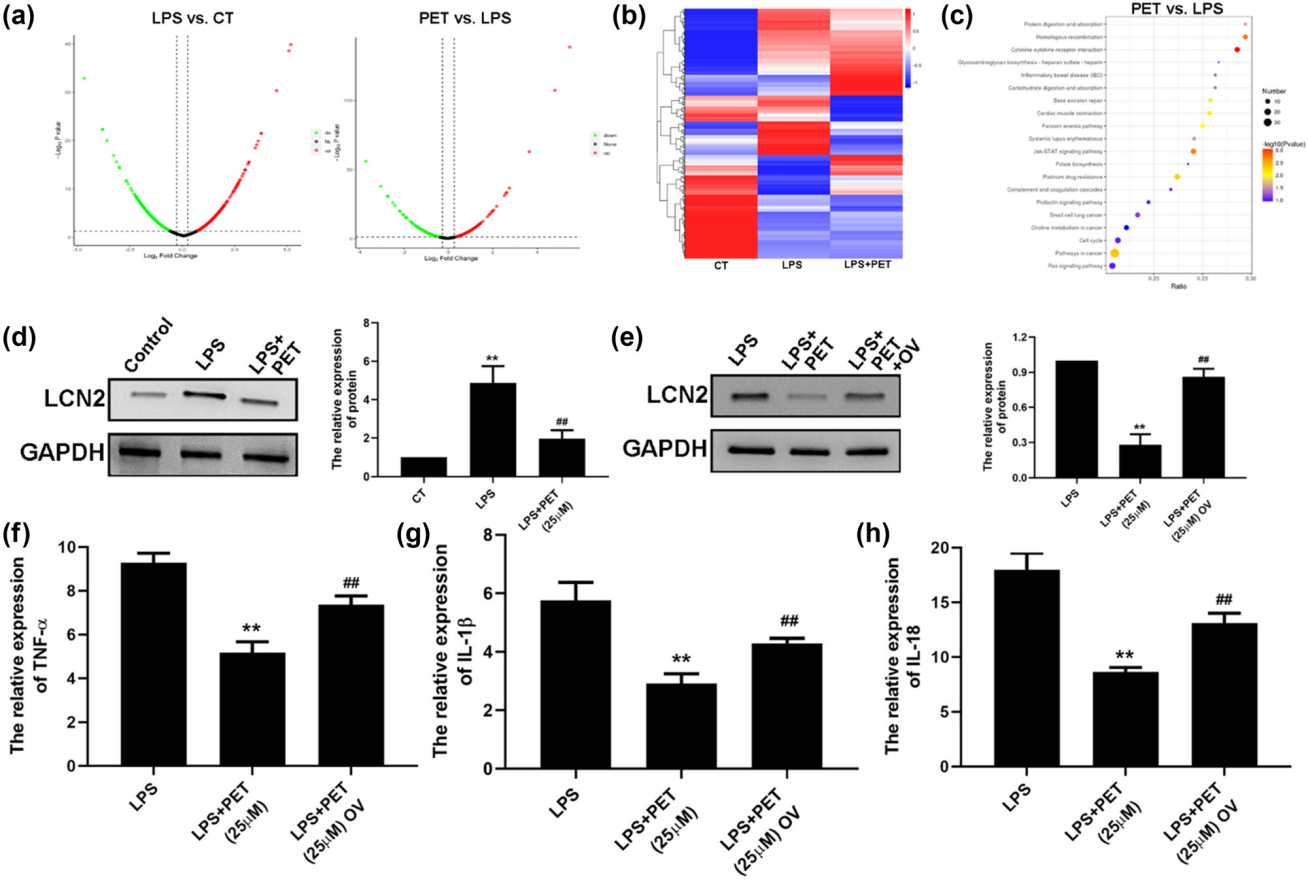
PET attenuated LPS-induced LCN2 upregulation in BV-2 cells. (a) Volcano plots of DEPs among three groups. (b) Heatmap analysis of DEPs among three groups. (c) KEGG analysis of DEPs between PET and LPS groups. (d) The effect of PET (25 µM) on LCN2 in BV2 cells upon LPS treatment (0.1 µg/mL) was assessed by Western blot analysis. **p < 0.01 vs CT, ^##^ *p* < 0.01 vs LPS (0.1 µg/mL). CT: the control group. (e) The effect of LCN2 overexpression in BV2 cells was assessed by Western blot analysis. (f) The effect of LCN2 overexpression on TNF-α expression in BV2 cells upon the indicated treatment was assessed by ELISA assay. (g) The effect of LCN2 overexpression on IL-1β expression in BV2 cells upon the indicated treatment was assessed by ELISA assay. (h) The effect of LCN2 overexpression on IL-18 expression in BV2 cells upon the indicated treatment was assessed by ELISA assay. **p < 0.01 vs LPS (0.1 µg/mL), ^##^ *p* < 0.01 vs LPS (0.1 µg/mL) + PET (25 µM).

To determine the anti-inflammatory target of PET upon LPS stimulation, iTRAQ-based proteomic analysis was conducted in BV2 cells. As shown in Figure 2a–c, there were 7,936 differentially expressed proteins (DEPs) identified in the LPS vs CT groups, and 7,063 DEPs identified in the PET vs LPS groups, among which, LCN2 was one of the most dysregulated proteins. As shown in Figure 2d, western blots analysis showed the consistent results compared to proteomics. As shown in Figure 2e–h, LCN2 overexpression was conducted, and which reversed the anti-inflammatory effect of PET in LPS‑treated BV-2 cells. The data indicated that LCN2 was the target of PET in LPS‑treated BV-2 cells.

### 3.3 PET reduces LCN2 expression by relocating and inactivating NF-κB

**Figure 3:**
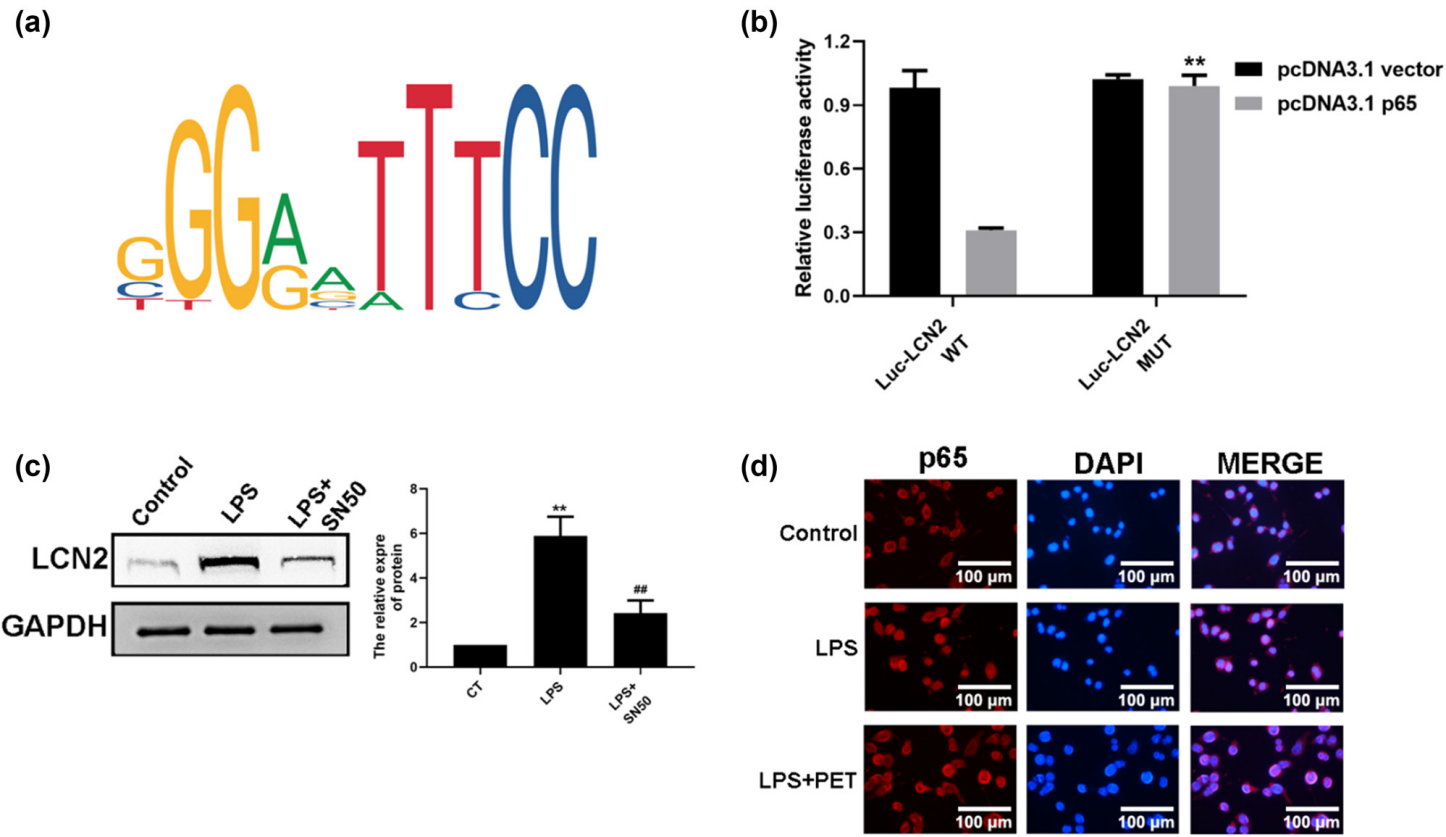
PET reduced LCN2 expression by relocating and inactivating NF-κB (p65). (a) Predicted p65 binding motifs and sequences from the JASPAR website. (b) p65 binding to LCN2 promoter were evaluated by dual luciferase reporter assay. **p < 0.01 vs Luc-LCN2 WT. (c) The effect of NF‑κB inhibitor SN50 (10 µg/mL) on LCN2 expression in BV2 cells was assessed by western blot analysis. (d) The effect of PET on nucleus translocation and activation of NF-κB (p65) in BV2 cells was assessed by immunofluorescence analysis. **p < 0.01 vs CT, ^##^ *p* < 0.01 vs LPS (0.1 µg/mL). CT: the control group.

To determine whether the inhibitory effect of PET on LCN2 expression was associated with regulation of transcription factor, the JASPAR database and dual-luciferase reporter assay were conducted. As shown in Figure 3a and b, based on JASPAR database analysis, NF-κB (p65) was predicted to be the up-regulatory transcription factor of LCN2, and which was verified by results from dual-luciferase reporter assay. As shown in Figure 3c, NF‑κB inhibitor SN50 decreased LCN2 expression in LPS-treated BV2 cells, confirming NF‑κB to be the transcription factor of LCN2. As shown in Figure 3d, nucleus translocation and activation of NF-κB were observed in BV2 cells with LPS stimulation. However, PET pre-treatment notably attenuated the effect of LPS by re-located NF-κB to cytoplasm.

### 3.4 NF-κB activation attenuates PET’s anti-inflammatory effects

**Figure 4:**
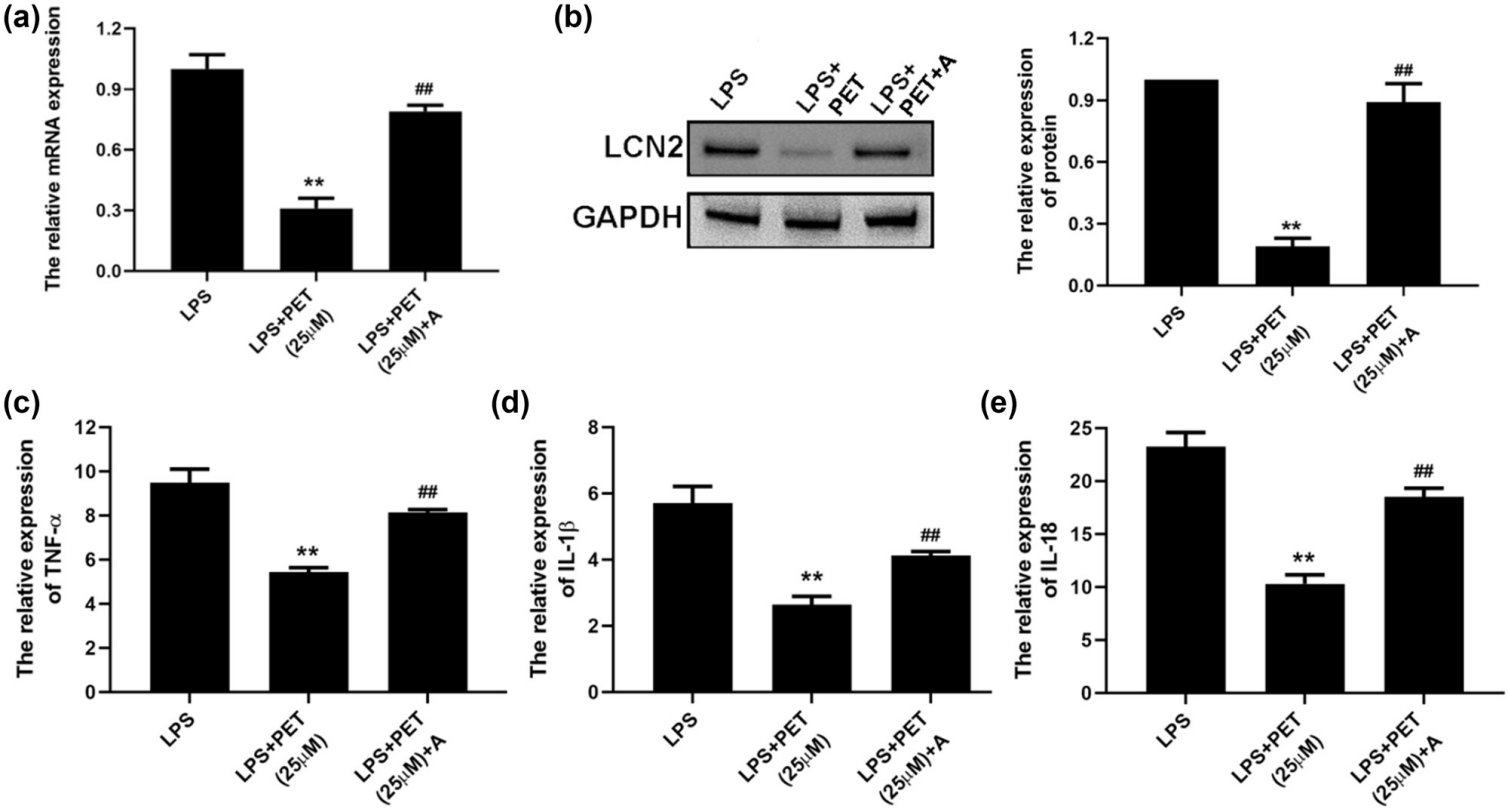
NF-κB activation attenuated PET’s anti-inflammatory effects in BV-2 cells. (a) The effect of NF-κB activator 1 (1 µM) on the mRNA level of LCN2 in BV2 cells was assessed by real-time PCR analysis. (b) The effect of NF-κB activator 1 (1 µM) on the protein level of LCN2 in BV2 cells was assessed by western blot analysis. (c) The effect of NF-κB activator 1 (1 µM) on TNF-α expression in BV2 cells upon indicated treatment was assessed by ELISA assay. (d) The effect of NF-κB activator 1 (1 µM) on IL-1β expression in BV2 cells upon indicated treatment was assessed by ELISA assay. (e) The effect of NF-κB activator 1 (1 µM) on IL-18 expression in BV2 cells upon indicated treatment was assessed by ELISA assay. **p < 0.01 vs LPS (0.1 µg/mL), ^##^ *p* < 0.01 vs LPS (0.1 µg/mL) + PET (25 µM). A: NF-κB activator 1.

To determine the role of NF‑κB in the PET’s anti-inflammatory effects on LCN2 expression and cytokines secretion, the activator of NF‑κB (NF-κB activator 1) was used in BV2 cells before the indicated treatment. As shown in Figure 4a and b, NF-κB activator 1 (1 µM) significantly up-regulated LCN expression both in mRNA and protein levels. As shown in Figure 4c–e, NF-κB activator 1 (1 µM) further reversed the anti-inflammatory effect of PET in LPS‑treated BV-2 cells by up-regulating cytokines secretion. The data indicated that the regulatory effect of PET on LCN2 was associated with the regulation of the NF‑κB signaling in LPS‑treated BV2 cells.

### 3.5 PET inhibits NF-κB activation by regulating *O*-GlcNAc modification

**Figure 5:**
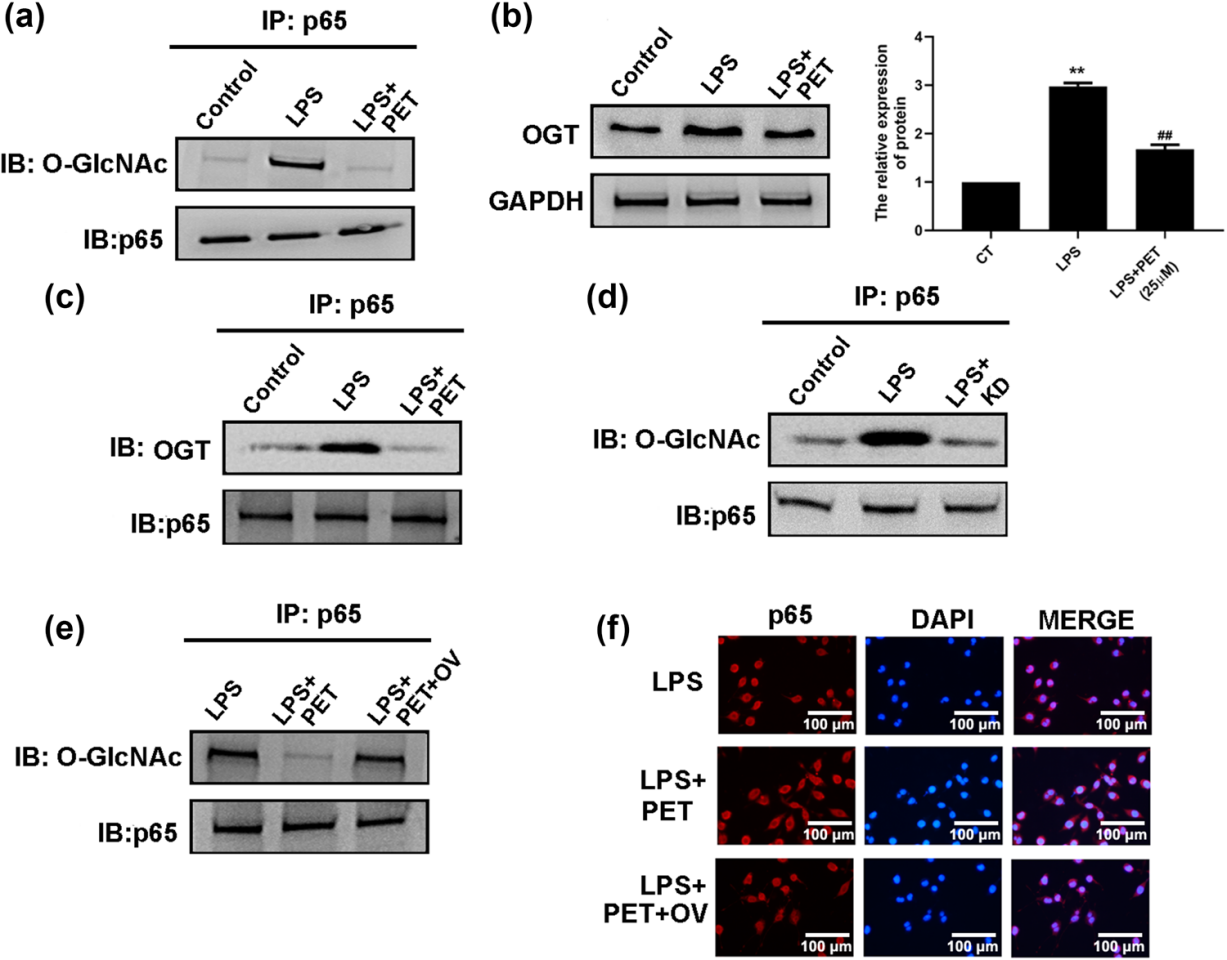
PET inhibited NF-κB activation by regulating *O*-GlcNAc modification. (a) The effect of PET on *O*-GlcNAc modification of p65 was assessed by Co-IP. (b) The effect of PET on the protein level of OGT in BV2 cells was assessed by western blot analysis. **p < 0.01 vs CT, ^##^ *p* < 0.01 vs LPS. CT: the control group. (c) The effect of PET on the interaction of OGT and p65 was assessed by Co-IP. (d) The effect of OGT knockdown on *O*-GlcNAc modification of p65 was assessed by Co-IP. (e) The effect of OGT overexpression on *O*-GlcNAc modification of p65 was assessed by Co-IP. (f) The effect of OGT overexpression on nucleus translocation and activation of NF-κB in BV2 cells was assessed by immunofluorescence analysis. OV: OGT overexpression.

To determine the regulatory mechanism of PET on NF-κB signaling, the *O*-GlcNAc modification of p65 was assessed in BV2 cells. As shown in Figure 5a, LPS stimulation notably induced the *O*-GlcNAc modification of p65; however, PET pre-treatment markedly attenuated this effect. As shown in Figure 5b, *O*-GlcNAc transferase (OGT) was up-regulated in LPS-treated BV2 cells and down-regulated in LPS + PET-treated BV2 cells, which was consistent to the change in *O*-GlcNAcylation of p65 in BV2 cells. As shown in Figure 5c and d, OGT overexpression partly reversed the *O*-GlcNAc modification of p65 in LPS + PET-treated BV2 cells. The data indicated that the regulatory effect of PET on NF-κB signaling were associated with the *O*-GlcNAc modification of p65 in LPS‑treated BV2 cells.

## 4 Discussion

PET is one of the most abundant natural anthocyanins, widely found in *Morus nigra*, *Berberis vulgaris*, and *Ipomea batatas*. A variety of bioactivities of PET are reported, such as anti-inflammation, anti-cancer, and renal-protective effects [19,25,26]. In this study, PET was found to attenuate LPS-induced inflammation in BV-2 cells and PRMC, and which was achieved by reducing LCN2 and inactivating NF-κB. In fact, LPS-induced microglial models are commonly used to simulate retinal inflammation in DR, and our *in vitro* experiments may suggest potential anti-inflammatory effect of PET in this disease.

LCN2 is a member of the adipokine proteins and regulates several cellular processes in eukaryotes [27]. LCN2 contributes to chronic inflammation, making it to be an important factor in several diseases including cancers, diabetes, obesity, and multiple sclerosis [28,29,30]. Gupta et al. reported that increased LCN2 in the retinal pigment epithelium cells drives dry age-related macular degeneration pathology by regulating macroautophagy/autophagy and inducing STING1-mediated inflammasome activation [31]. Wen et al. reported that the expression of LCN2 in microglia/macrophages of DR is up-regulated by Kdm6a, and which promotes and impairs glycolysis progression in photoreceptor cells [32]. In this study, iTRAQ-based proteomic analysis and western blot analysis confirmed that LCN2 was upregulated in LPS group and downregulated in LPS + PET group, and LCN2 overexpression reversed the protective effect of PET in LPS-treated BV2 cells. The above data indicated that LCN2 was the target of PET in attenuating the inflammatory status of retinal microglia. Then, to investigate the upstream transcription factor, the JASPAR database and dual-luciferase reporter assay were conducted and revealed that the NF-κB was the upregulatory transcription factor of LCN2.

NF-κB is a transcription factor involved in various cellular processes, especially in mediating inflammatory responses [33,34]. In resting cells, NF-κB is retained in the cytoplasm through its association with IκB proteins, which mask the nuclear localization signals (NLS) within the p65 subunit. Upon stimulation with diverse cues such as inflammatory cytokines or cellular stress, IκB undergoes phosphorylation and subsequent proteasomal degradation, enabling p65 to undergo nuclear translocation. The p65 subunit contains the major transcriptional activation domain (TAD), particularly the C-terminal region (TA1/TA2), which is essential for binding to DNA and activating the transcription of NF-κB-responsive genes [35]. In BV2 microglia cells, decreased release of LCN2 mediated by PET was associated with significant inhibition of NF-κB nuclear translocation, and NF-κB activator 1 could reverse the protective effect of PET on LCN expression and inflammation, confirming that the drug interfered the production of inflammatory factors by regulating NF-κB signaling pathway. *O*-linked-β-*N*-acetylglucosamine (*O*-GlcNAcylation) is a distinctive posttranslational protein modification targeting serine or threonine residues, via *O*-GlcNAc transferase and *O*-GlcNAcase [36]. Dysregulation of *O*-GlcNAcylation in p65 contributes to many human diseases including diabetes [37]. *O*-GlcNAc transferase (OGT)-mediated *O*-GlcNAcylation can promote p65 phosphorylation, nuclear translocation, NF-κB transcriptional activity, and levels of NF-κB transcriptional targets TNF-α in microglia. Our data further confirmed that the regulatory effect of PET on NF-κB signaling was associated with the OGT-mediated *O*-GlcNAc modification of p65 in LPS‑treated BV2 cells. In addition, OGT overexpression partly reversed the *O*-GlcNAc modification of p65 in LPS + PET-treated BV2 cells. The data may indicate that PET affects OGT activity by downregulating OGT expression, but not directly affecting OGT activity, and further study needs to clarify this mechanism.

In conclusion, this study demonstrated the protective effect and detailed mechanism of PET on LPS‑treated retinal microglia, which was achieved through the suppression of LCN2 expression and the activation of NF-κB signaling upon LPS treatment. In the context of DR, LPS-induced microglial models are commonly used to understand the complex molecular and cellular mechanisms underlying retinal inflammation, and then which was used in this study to preliminarily evaluate the effect of PET; however, there are still numerous questions regarding the *in vivo* anti-inflammatory effect of PET in DR animal model. In addition, the potential side effects (off-target effects) and pharmacokinetic properties of PET still need to be investigated in further study. Therefore, the present and a future *in vivo* study may contribute to the pharmaceutical potential of PET and its derivatives in therapy for DR.
